# Psychometric properties of the Brief Pain Inventory among patients with osteoarthritis undergoing total hip replacement surgery

**DOI:** 10.1186/1477-7525-8-148

**Published:** 2010-12-09

**Authors:** Heidi Kapstad, Berit Rokne, Knut Stavem

**Affiliations:** 1 Oslo University College, Faculty of Nursing Education, Oslo, Norway; 2 Department of Public Health and Primary Health Care, University of Bergen, Bergen, Norway; 3 Buskerud University College, Department of Health Sciences, Drammen, Norway; 4 Department of Pulmonary Medicine, medical Division, Akershus University Hospital, Lørenskog, Norway; 5 Helse-Øst Health Services Research Centre, Lørenskog, Norway; 6 Faculty of Medicine, University of Oslo, Oslo, Norway

## Abstract

**Background:**

Pain is a cardinal symptom of osteoarthritis (OA) of the hip and important for deciding when to operate. This study assessed the internal consistency reliability, validity and responsiveness of the Brief Pain Inventory (BPI) among patients with OA undergoing total hip replacement (THR).

**Methods:**

We prospectively included 250 of 356 patients who were accepted to the waiting list for primary THR surgery. All participants responded to the BPI, WOMAC and SF-36 at baseline and 1 year after surgery.

**Results:**

Internal consistency reliability (Cronbach's α) was >0.80 for the BPI, the WOMAC and five of the eight SF-36 scales The pattern of associations of the two BPI scales with corresponding and non-corresponding scales of the WOMAC and SF-36 largely supported the construct validity of the BPI. The responsiveness indices for change from baseline to 1 year after THR ranged from 1.52 to 2.05 for the BPI scales, from 1.69 to 2.84 for the WOMAC scales, and from 0.25 (general health) to 2.77 (bodily pain) for the SF-36 scales.

**Conclusions:**

The BPI showed acceptable reliability, construct validity and responsiveness in patients with OA undergoing THR. BPI is short and therefore is easy to use and score, though the instrument offers few advantages over and duplicates scales of more comprehensive instruments, such as the WOMAC and SF-36.

## Background

Primary total hip joint replacement (THR) is an effective intervention for severe osteoarthritis (OA) of the hip that relieves the patients' pain, increases physical functioning, and improves health related quality of life (HRQoL). Previously, evaluation of surgery for OA of the hip has typically been done with functional scoring systems, such as the Harris Hip score [1-3].

During the last decade patient-reported outcomes, such as HRQoL, have gained importance in the assessment of outcome after surgery for OA of the hip [4-7]. The two most commonly used questionnaires to assess the outcome of hip surgery are the generic Medical Outcomes Short Form 36 Health Survey (SF-36) and the more disease-specific Western Ontario and McMaster Universities Osteoarthritis Index (WOMAC) [4-6,8-11].

Pain is a cardinal symptom of OA of the hip and is probably the most important variable for deciding whether to operate or not. Therefore, questionnaires specifically developed for the assessment of pain can potentially complement the WOMAC and the SF-36 among patients with OA, or possibly be more sensitive to change. The Brief Pain Inventory (BPI) is a self-administered questionnaire developed to assess pain and the impact of pain [12]. It was developed for use in cancer pain, but has also been used in other chronic pain conditions [13-18].

The reliability, validity and responsiveness of the BPI, or a shortened version of it, after drug interventions, have recently been reported in unspecified patients with OA [15,16], but its psychometric properties have not been documented in homogeneous samples of patients with OA of the hip, or in surgical intervention for OA of the hip. If the BPI is to be used in such a setting, it is important to document the properties of the questionnaire. In the present study we wanted to assess the psychometric properties of the BPI in patients with OA of the hip undergoing THR.

## Materials and methods

### Subjects and study design

**Figure 1:**
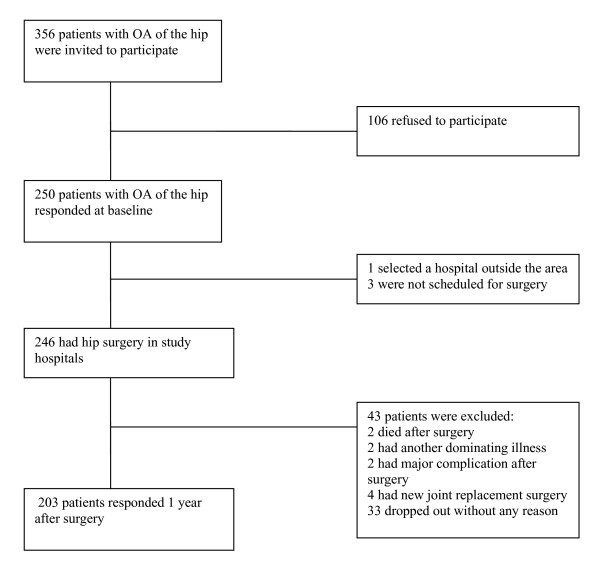
**Flow chart**.

The study was a prospective multi-center study in six hospitals in three Norwegian counties. We included consecutive patients >18 years old, who were accepted to the waiting list for primary THR surgery and had satisfactory proficiency of the Norwegian language to respond to questionnaires. Between June 2003 and June 2004, 356 patients were invited, and 250 (70%) accepted to participate and responded at baseline (Figure 1).

In each participating hospital a project contact in the orthopedic unit identified patients fulfilling the inclusion criteria. All participants responded to a package of questionnaires at (i) acceptance to the waiting list for surgery (baseline), and (ii) 1 year after surgery. At baseline, we mailed the questionnaire to the subjects shortly after an ambulatory visit.

Among the 250 baseline respondents, we excluded those that had new joint replacement surgery (n = 4), were not scheduled for surgery (n = 3), had severe complication after surgery (n = 2), had another dominating disease (n = 2), had died (n = 2) or chose another hospital for surgery (n = 1). The remaining 236 baseline respondents received questionnaires 1 year after surgery, of whom 203 responded to the questionnaire (80% of all baseline respondents). In the planning and establishing of this study sample size was calculated. The study was powered detect a change in HRQoL of 0.5 SD, with power 0.9, and 5% significance level. In paired analysis, this would require a sample size of at least 43 patients for analysis. To account for possible loss to follow-up and comparisons of changes in subgroups, we chose to include about 250 patients.

The Regional Committee for Medical Research Ethics and the Norwegian Social Science Data Services approved the study.

### Questionnaires

At baseline, patients completed a questionnaire that comprised data on gender, age, marital status, cohabitation, education level, employment status, type of surgery, duration of pain in the joint, and number of years with ambulation problems. In addition, patients completed the Brief Pain Inventory (BPI) [12], the WOMAC [19,20], and the SF-36 [21-23] questionnaires.

### The brief pain inventory

The BPI is a short, self-administered questionnaire with 11 items, which was designed to evaluate the intensity of, and the impairment caused by, pain. Originally, the BPI was developed to evaluate cancer pain, but it has been shown to be a valid and reliable instrument for chronic non-cancer pain [13,14,17]. All BPI items are scored using rating scales. Four items measure pain intensity (pain now, average pain, worst pain, and least pain) using 0 ("no pain") to 10 ("pain as bad as you can imagine") numeric rating scales, and seven items measure the level of interference with function caused by pain (general activity, mood, walking ability, normal work, relations with other persons, sleep, and enjoyment of life) using 0 (no interference) to 10 (complete interference) rating scales.

The items are aggregated into two dimensions, (1) Pain severity index, using the sum of the four items on pain intensity, and (2) Function interference index, using the sum of the seven pain interference items [18,24,25]. Missing values were handled as recommended by the developers of the BPI [26]. All four severity items must been completed for aggregating a pain severity index. The function interference index is scored as the mean of the items scores multiplied by seven, given that more than 50%, or four of seven, of the items have been completed [26]. We used the Norwegian translation of the BPI, which has documented reliability and validity [24].

### The womac osteoarthritis index

The WOMAC is a validated and sensitive instrument that can detect clinically important changes following a variety of interventions for OA [19]. It is a three-dimensional, disease-specific, and self-administered instrument [19,20] that consists of 24 items that evaluate pain (five items), stiffness (two items), and overall level of physical function (17 items). Items are rated using one of five responses (0 = none, 1 = mild, 2 = moderate, 3 = severe, 4 = extreme). The item scores are aggregated to three subscale scores, pain, stiffness, and physical function, which are calculated as the mean of the item scores in each dimension. Finally, all subscales were recoded to 0-10 scales to ease interpretation, where 10 represents maximal problems and 0 no problems [27].

For this study, patients were asked to respond to each item in relationship to the hip joint that was to be replaced and to respond in relationship to the past 48 hours. We used the Norwegian Likert scale version 3.1 [28,29].

### Medical outcomes study short form (SF-36)

The SF-36 questionnaire consists of 36 items that evaluate eight conceptual domains of HRQoL: general health (GH), physical functioning (PF), mental health (MH), role limitations - physical (RP), role limitations- emotional (RE), vitality (VT), bodily pain (BP), and social functioning (SF) [22]. The SF-36 is a widely used measure of HRQoL with documented validity and reliability in various languages and populations [22,23]. This instrument has previously been used in patients with OA [4-6,8-10,30-33]. The Norwegian translation of the standard SF-36 version 1.1 was used and scored on a 0-100 scale, with higher scores indicating a better HRQoL [34].

### Statistical analysis

Descriptive statistics are presented using the mean (SD) or numbers (percentages). Groups were compared using the chi-square test, independent samples t-test, or Mann-Whitney U test, where applicable. Internal consistency reliability for the BPI, WOMAC and SF-36 scales at baseline was assessed using Cronbach's coefficient alpha [35]. A commonly accepted requirement for internal consistency reliability is that it should be at least 0.70 [36]. A floor effect occurs when a high proportion of the respondents grade themselves at the minimum score and a ceiling effect when a high proportion of the respondents score at the maximum of the instrument. Because the patients' perceived pain and HRQoL scores were expected to be very different before and after surgery, we estimated the floor and ceiling effect for the BPI, WOMAC and SF-36 at baseline and 1 year after surgery. Such effects may limit changes in scores in one direction, hence limiting an instrument to capture changes.

Construct validity of the BPI was assessed by Pearson's product-moment correlation coefficient between the BPI scale scores and scores on the WOMAC and SF-36 scales, using the baseline data in this study. Before this analysis, based on assessment of the content of the items on the scales, we hypothesized that (1) the Bodily pain (BP) scale of the SF-36 and the Pain scale of the WOMAC would represent similar constructs as the Pain severity index of the BPI, and (2) the SF-36 BP, PF and RP scales, and all three WOMAC scales would correspond with the Function interference index of the BPI. A finding of higher intercorrelations (r > 0.4) between subscales assessing similar constructs, compared with subscales assessing dissimilar constructs, would support the construct validity of the BPI.

Responsiveness was evaluated by longitudinal assessment of patients undergoing THR, investigating if the instruments were sensitive to change following the intervention. Responsiveness was further investigated in categories of respondents, stratified according to the response on an item on change in overall health during the past year. We used item two on the SF-36 questionnaire as the rating of overall change: "Compared to 1 year ago, how would you rate your health in general now? (1 = much better, 2 = somewhat better, 3 = about the same, 4 = somewhat worse, 5 = much worse)". The respondents were categorized as having a better (1 to 2), unchanged (3) or worse (4 to 5) health status [36,37]. In assessment of correlations between indices and responsiveness, we standardized the analysis by only including respondents that had valid scores on all scales of the three questionnaires (n = 161).

To assess the magnitude of the responsiveness, we calculated the effect size (ES), standardized response mean (SRM), and Responsiveness Index (RI). ES= (mean 1 year after - mean at baseline)/SD baseline, SRM= (mean 1 year after - mean at baseline)/SD difference, and RI= (mean 1 year after - mean at baseline)/SD of change scores in patients with unchanged health status [36-38].

Data were analyzed using SPSS for Windows version 13.0 (SPSS Inc., Chicago, Ill.). We chose a 5% significance level using two-sided tests.

## Results

In total, 203 patients completed the study 1 year after THR, 143 (70%) were female. The patients completing the study had a mean age of 69 years (SD 10), 67% were married/cohabiting, and 61% were retired. The respondents and non-respondents did not differ in baseline characteristics, though the non-respondents tended to be slightly older and more of them were retired than the respondents (Table 1).

**Table 1:** Patient characteristics at baseline for respondents included in the analysis 1 year after hip joint replacement surgery and those excluded after baseline response, mean (SD) unless otherwise stated.

	Included	Excluded	p
*N*	*203*	*47*	
Female sex, number (%)	143 (70)	35 (74)	0.58
Age, years	68.7 (9.9)	71.7 (8.4)	0.06
Marital status, number (%)			0.37
Married	135 (67)	31 (66)	
Unmarried	11 (5)	0 (0)	
Separated/divorced	24 (12)	6 (13)	
Widowed	33 (16)	10 (21)	
Employment, number (%)			0.07
Retired	123 (61)	35 (76)	
Disability pension	25 (12)	4 (9)	
Sick leave	17 (8)	5 (11)	
Full or part time employed	38 (19)	2 (4)	
Educational level, number (%)			0.19
Primary school	49 (25)	17 (37)	
Secondary school	85 (42)	19 (41)	
University < 4 years	39 (19)	4 (9)	
University ≥4 years	28 (14)	6 (13)	
Comorbidity, number (%)	***(N = 168)***	***(N = 42)***	
Cardiovascular	33 (20)	9 (21)	0.47
Pulmonary	21 (13)	7 (17)	0.31
Diabetes	5 (3)	3 (7)	0.20
Cancer	21 (13)	6 (14)	0.46
Skin diseases	21 (13)	2 (5)	0.12
Gastrointestinal	26 (16)	5 (12)	0.38
Psychiatric	18 (11)	3 (7)	0.36
Other	28 (17)	5 (12)	0.31
Duration of pain prior to surgery, years	6.3 (6.7)^1^	6.5 (6.1) ^2^	0.82
Waiting time, days^3^	68.6 (54.3)	65.6 (58.8)^4^	0.75

^1^n = 181, ^2^n = 42, ^3^from baseline to surgery, ^4^n = 40

At baseline, internal consistency reliability, as assessed with Cronbach's α, was >0.80 for the BPI pain severity index and function interference index, the WOMAC and the SF-36 subscales except the pain and stiffness subscale on the WOMAC, the BP and GH scales of the SF-36 (0.79, 0.70, 0.68 and 0.69, respectively) (Table 2).

**Table 2:** Psychometric properties at baseline and 1 year after primary hip joint replacement

		Baseline				1 year after surgery			
			
	Number of items	N	% Floor^5^	%Ceiling^6^	Cronbach's α^7^	N	% Floor^5^	%Ceiling^6^	Cronbach's α^7^
**Brief Pain Inventory**									
Pain severity index^1^	4	232	0	1	0.88	200	21	0	0.91
Function interference index^2^	7	234	0.4	1.3	0.87	191	24	0	0.95
**WOMAC **^3^									
Pain	5	247	0.4	1.6	0.79	203	37	0	0.87
Stiffness	2	247	0.8	4.0	0.70	203	28	0	0.84
Physical function	17	248	0	0	0.93	202	9	0	0.96
**SF-36 Scale**^4^									
Physical functioning	10	248	4	0	0.82	201	1	4	0.92
Role-physical	4	247	82	4	0.84	197	37	34	0.89
Bodily pain	2	250	6	0	0.68	200	0.5	27	0.87
General health	5	240	0.8	3	0.69	196	0	7	0.81
Vitality	4	242	3	0	0.82	199	0	4	0.86
Social functioning	2	250	4	18	0.82	202	0.5	56	0.89
Role-emotional	3	242	42	40	0.91	198	23	60	0.89
Mental health	5	240	0	7	0.85	198	0	12	0.78

^1 ^Range 0 - 40 (no pain, pain as bad you can image)

^2 ^Range 0 - 70 (does not interfere, interferes completely)

^3 ^Range 0 - 10 (no problem, maximum problem)

^4 ^Range 0 - 100 (poor health status, maximal health status)

^5 ^% scoring worst possible value

^6 ^% scoring best possible value

^7 ^Internal consistency reliability

None of the BPI indices had marked floor or ceiling effects at baseline, however, at 1 year after THR the BPI pain severity index and function interference index showed a floor effect, but none had signs of a ceiling effect. On the WOMAC subscales at baseline, floor and ceiling effects were minor. One year after THR, the floor effect was larger on all three WOMAC subscales, but most marked on the pain and stiffness subscales. At baseline, floor and ceiling effects on the SF-36 subscales were most marked on the RP and RE scales, and 1 year after THR there was marked ceiling effects on the SF and RE scales (Table 2).

In total, 161 of the 203 patients had valid dimension scores on all three questionnaires at baseline and 1 year after THR. The correlations between the two BPI scales and scales of the WOMAC and SF-36 partially supported our hypotheses (Table 3). Correlation of the BPI pain severity index with the pain subscale on the WOMAC and BP on the SF-36 were high, in line with hypothesis (1). In addition the physical function scale on the WOMAC was highly correlated with the BPI pain severity index (r = 0.57). Further the BPI function interference index scores and the subscales on the WOMAC except stiffness were highly correlated in accordance with hypothesis (2). The results indicated moderate to high correlations of the BPI function interference index with BP and PF scales, as hypothesized, but low correlation with the RP scale. Further, the correlations of the BPI function interference index with the VT, SF and MH scales were moderate to high. The correlations between hypothesized non-corresponding items were lower (Table 3).

**Table 3:** Pearson's product-moment correlation coefficient between the Brief Pain Inventory pain (BPI) severity index and function interference index with subscales of the WOMAC and the SF-36 questionnaires for patients with OA of the hip at baseline (N = 161)

	Pain Severity Index	Function Interference Index
**WOMAC**		
Pain	0.66**	0.57**
Stiffness	0.26**	0.33**
Physical function	0.57**	0.63**
**SF-36 Scale**		
Physical functioning	-0.38**	-0.51*'
Role-physical	-0.22**	-0.32**
Bodily pain	-0.58**	-0.65**
General health	-0.27**	-0.37**
Vitality	-0.39**	-0.57**
Social functioning	-0.45**	-0.63**
Role-emotional	-0.19*	-0.40**
Mental health	-0.41**	-0.67**

**correlation is significant at the 0.01 level

* correlation is significant at the 0.05 level

Hypothesized moderate to high correlations are boldfaced

For the BPI, the responsiveness indices (ES, SRM and RI) for change from baseline to 1 year after THR for the total sample were large, with minimum values of 1.57 for the pain severity and 1.52 for the function interference index (Table 4). Also on the WOMAC scales the responsiveness indices on the three subscales were large, minimum values ranging from 1.69 to 2.84.

**Table 4:** Responsiveness indices (Effect Size (ES), Standardized Response Mean (SRM) and Responsiveness Index (RI)), for change from baseline to 1 year after primary hip joint replacement surgery (N = 161).

	ES	SRM	RI
**Brief Pain Inventory**			
Pain severity index^1^	1.57	1.61	2.03
Function interference index^2^	1.71	1.52	2.05
**WOMAC**^3^			
Pain	-2.69	-2.52	-2.84
Stiffness	-2.28	-1.75	-1.69
Physical function	-2.61	-2.33	-2.35
**SF-36 Scale**^4^			
Physical functioning	2.17	1.54	1.85
Role-physical	1.46	0.95	0.94
Bodily pain	2.77	1.78	1.69
General health	0.24	0.25	0.29
Vitality	0.77	0.82	1.10
Social functioning	0.80	0.85	0.78
Role-emotional	0.49	0.50	0.36
Mental health	0.42	0.52	0.49

^1 ^Range 0 - 40 (no pain, pain as bad you can image)

^2 ^Range 0 - 70 (does not interfere, interferes completely)

^4 ^Range 0 - 100 (poor health status, maximal health status)

On the eight SF-36 scales the responsiveness indices showed more variation. For the PF, RP and BP scales the responsiveness indices were all above 0.94, for VT and SF scales they ranged from 0.77 to 0.85, except for the RI which was 1.10 for the VT. The remaining SF-36 scales, GH, RE and MH were less responsive, with responsiveness indices from 0.24 to 0.52.

In analysis of responsiveness in groups stratified according to the rating of overall health change: 133 reported better, 28 unchanged or worsened overall health than 1 year before. Those that reported an improvement in the rating of overall health change over 1 year had large values on all responsiveness indices on the pain severity index and function interference index of the BPI and all the subscales of the WOMAC and the SF-36, except GH, RE and MH, with values >0.80. All responsiveness indices for this group were larger than for the groups with unchanged or worsened overall health (Table 5).

**Table 5:** Responsiveness indices (Effect Size (ES), Standardized Response Mean (SRM) and Responsiveness Index (RI)), for change from baseline to 1 year after hip joint replacement surgery, according to perceived global change.

	Improved (n = 133)			Unchanged or worsened (n = 28)		
	**ES**	**SRM**	**RI**	**ES**	**SRM**	**RI**
	
**Brief Pain Inventory**						
Pain severity index^1^	1.70	1.71	2.17	1.00	1.36	1.39
Function interference index^2^	1.80	1.56	2.16	1.27	1.40	1.56
**WOMAC**^3^						
Pain	-2.98	-2.82	-3.01	-1.61	-1.84	-2.07
Stiffness	-2.46	-1.90	-1.76	-1.56	-1.25	-1.33
Physical function	-2.96	-2.75	-2.53	-1.35	-1.53	-1.50
**SF-36 Scale**^4^						
Physical functioning	2.36	1.68	2.01	1.35	1.53	1.50
Role-physical	1.50	1.02	1.01	1.26	1.19	1.10
Bodily pain	2.87	1.95	1.80	2.16	1.31	1.17
General health	0.37	0.40	0.43	-0.36	-0.41	-0.42
Vitality	0.85	0.88	1.19	0.46	0.58	0.66
Social functioning	0.93	0.97	0.84	0.40	0.50	0.46
Role-emotional	0.54	0.60	0.40	0.23	0.19	0.18
Mental health	0.51	0.69	0.59	0.01	0.01	0.01

^1 ^Range 0 - 40 (no pain, pain as bad you can image)

^2 ^Range 0 - 70 (does not interfere, interferes completely)

^3 ^Range 0 - 10 (no problem, maximum problem)

^4 ^Range 0 - 100 (poor health status, maximal health status)

For those with unchanged or worsened overall health the responsiveness indices indicated an improvement in pain and HRQoL, with large responses on the BPI pain severity and function interference indices, the three WOMAC subscales, and for some of the SF-36 scales most related to physical health (PF, RP and BP). For the other SF-36 scales the effects were moderate (VT and SF), small (RE) or unchanged (MH). For the GH scale, the responsiveness indices changed in the opposite direction, suggesting a slight deterioration.

## Discussion

In the present study we have documented the psychometric properties of the BPI in patients with OA of the hip, using standard methodology for assessing internal consistency reliability, validity and responsiveness. The BPI showed satisfactory internal consistency reliability with Cronbach's alpha >0.80 in assessment of pain and the impact of pain [36]. The pattern of observed correlations between subscales of the BPI measuring constructs similar to the WOMAC and SF-36 questionnaires generally were in line with expectations, thereby supporting the construct validity of the BPI scales in this setting. Further, the study has shown that the BPI also was responsive and detected change in pain and the impact of pain from before to 1 year after THR in a homogenous sample of patients with OA. The responsiveness of the BPI pain severity and function interference indices were at the level of the three WOMAC subscales and the PF, RP and BP scales of the SF-36, and in line with previous studies using the WOMAC [11,39].

The internal consistency reliability for the BPI pain severity and function interference indices was in line with previous reports in patients with chronic non-malignant pain [14], OA [16], or undergoing cardiac surgery [18]. The high Cronbach's alpha in both dimensions of the BPI suggests that both indices are sufficiently unidimensional to permit scoring of the items as two composites.

The pattern of associations between the two BPI scales and corresponding and non-corresponding scales of the WOMAC and SF-36 largely supported the construct validity of the BPI. Our hypothesis was fairly crude and based on the judgment of items and scales, which cannot be expected to exactly capture all associations. At the same time it suggests that there is some overlap between the instruments, and that the BPI scales to some extent duplicate the BP scale of the SF-36 and the WOMAC pain subscale.

A previous study stated that 15% is a critical value for floor and ceiling effects [40]. In the present study, BPI subscale scores at baseline showed nearly no floor or ceiling effects. One year after THR, there was floor effects for both BPI subscales of 21% and 24%, respectively, and no ceiling effects. These results can be explained by the natural history of patients with OA that undergo THR; the lowest possible score is zero for a subject who refers to" no pain" for the pain severity index and "does not interfere" for the function interference index. For the WOMAC, the floor effect after THR was very large for the subscales pain and stiffness. These two subscales have fewer items than physical function. For the SF-36, the RP and RE scales had excessive floor and ceiling effects at baseline and 1 year after THR, and also the BP and SF scales presented excessive ceiling effects after THR, as in a previous study [11]. The large floor and ceiling effects may be related to the low number of possible values on these scales, as the RE, RP, BP and SF scales on the SF-36 have either few items or each item is scored on a binary scale.

The responsiveness of a measure is commonly appraised by comparing an observed change to another independent criterion, such as patient-perceived transition of health change [37]. In the present study, we used an item from the SF-36, which is not included in scoring of the SF-36 scales, where we categorized the respondents as having a better or unchanged/worse health status based on responses to a five point Likert scale. The stratified analysis according to rating of overall health change showed that the responsiveness indices (ES, SRM and RI) were large for the subscales on the WOMAC and the BPI pain severity and function interference indices and largest on both questionnaires among patients who reported improved health status. A previous study about responsiveness for the WOMAC and SF-36 after THR also reported good responsiveness on the WOMAC subscales and the physical domains on the SF-36 [11].

The results in the unchanged/worsened group seemed to be in the opposite direction of what would be expected, i.e. they suggested some improvement, but less than in the improved group. This may be caused by the crudeness of the rating of overall health change, in an intervention that for the majority of patients was very effective. Alternatively, it may be caused by recall bias, or other changes in health that were unrelated to OA or THR in this sample of elderly people with considerable comorbidity. Hence, the improvement with the disease-specific questionnaires may capture positive changes despite the patients' perspective of unchanged or worsened change in overall health.

The responsiveness of the BPI has previously not been reported among patients with OA undergoing THR. However, responsiveness of the BPI has been assessed in two previous studies: in patients undergoing cardiac surgery and patients with OA in a clinical trial of controlled-release oxycodone [16,18]. Both studies reported moderate to large responsiveness indices, supporting the responsiveness of the BPI.

Compared with the WOMAC and SF-36 the BPI is short, easy to use and score. In the present study there was little difference in missing change scores on the scales of the different instruments. Previous studies have shown that BPI is a feasible instrument for use among patients with pain. Pain is a cardinal symptom among patients with OA of the hip and an important indication for undergoing THR, Therefore, a systematic evaluation of self-reported pain and the impact of pain using the BPI could be a complement to assessment by the physician [41]. Further, changes in pain can be quantified in a meaningful way using the BPI and enable comparison of results between studies [41]. Because of its briefness, the BPI can possibly also be valuable in a daily diary context.

Some limitations of our study should be mentioned. We assessed construct validity by investigating the pattern of associations between the different scales. However, other forms of validity could have been assessed, such as known groups validity or criterion validity, but we thought we had no feasible variables for such analysis. We also did not assess the factor structure of the BPI, which could have been done with confirmatory factor analysis. A two-factor structure of the BPI has been reported in several previous studies [13,18,25,42], and we think this would contribute little to the paper. Responsiveness was in the present study assessed with an item from the SF-36 questionnaire, change in health in general, as a marker of overall health change. This was the best marker of overall health outcome that was available. We considered using responsiveness index which relates changes to an instrument's minimally important change. Because this is not reported for the BPI, we were unable to present this statistic. Further, we did not ask patients separate questions about changes in their physical health, mental health, pain or other components or symptoms and therefore cannot determine what components of health status were most important for the result. The sample size in the unchanged/worsened group was small, hence reducing the power of the study.

The BPI showed acceptable internal consistency reliability, construct validity and responsiveness in assessment of pain and impact of pain among patients with OA undergoing THR. We conclude that the BPI is a short instrument that can be used as an alternative or complement to more established instruments in this patient group, though the instrument offers few advantages over, and duplicates scales of, more comprehensive instruments, such as the WOMAC and SF-36.

## Competing interests

None of the authors have any personal or financial interest or relationship with other people or organizations that could inappropriately influence the work.

## Authors' contributions

All authors participated in planning and design of the study.

HK organised and supervised the data collection. HK and KS did the data analysis, drafted and revised the manuscript. BR reviewed and commented on the manuscript. All authors read and approved the final manuscript.

## References

[B1] Andersson G Hip assessment: a comparison of nine different methods J Bone Joint Surg Br 1972 54 621 625 4639438

[B2] Harris WH Traumatic arthritis of the hip after dislocation and acetabular fractures: treatment by mold arthroplasty. An end-result study using a new method of result evaluation J Bone Joint Surg Am 1969 51 737 755 5783851

[B3] Soderman P Malchau H Is the Harris hip score system useful to study the outcome of total hip replacement? Clin Orthop Relat Res 2001 189 197 10.1097/00003086-200103000-00022 11249165

[B4] Bachmeier CJ March LM Cross MJ Lapsley HM Tribe KL Courtenay BG Brooks PM A comparison of outcomes in osteoarthritis patients undergoing total hip and knee replacement surgery Osteoarthritis Cartilage 2001 9 137 146 10.1053/joca.2000.0369 11330253

[B5] Fortin PR Clarke AE Joseph L Liang MH Tanzer M Ferland D Outcomes of total hip and knee replacement: preoperative functional status predicts outcomes at six months after surgery Arthritis Rheum 1999 42 1722 1728 10.1002/1529-0131(199908)42:8<1722::AID-ANR22>3.0.CO;2-R 10446873

[B6] Jones CA Voaklander DC Johnstone DW Suarez-Almazor ME Health related quality of life outcomes after total hip and knee arthroplasties in a community based population J Rheumatol 2000 27 1745 1752 10914862

[B7] Mahomed NN Liang MH Cook EF Daltroy LH Fortin PR Fossel AH Katz JN The importance of patient expectations in predicting functional outcomes after total joint arthroplasty J Rheumatol 2002 29 1273 1279 12064846

[B8] Fortin PR Penrod JR Clarke AE St Pierre Y Joseph L Belisle P Liang MH Ferland D Phillips CB Mahomed N Tanzer M Sledge C Fossel AH Katz JN Timing of total joint replacement affects clinical outcomes among patients with osteoarthritis of the hip or knee Arthritis Rheum 2002 46 3327 3330 10.1002/art.10631 12483739

[B9] Mahon JL Bourne RB Rorabeck CH Feeny DH Stitt L Webster-Bogaert S Health-related quality of life and mobility of patients awaiting elective total hip arthroplasty: a prospective study CMAJ 2002 167 1115 1121 12427702 PMC134291

[B10] Salaffi F Carotti M Grassi W Health-related quality of life in patients with hip or knee osteoarthritis: comparison of generic and disease-specific instruments Clin Rheumatol 2005 24 29 37 10.1007/s10067-004-0965-9 15674656

[B11] Quintana JM Escobar A Bilbao A Arostegui I Lafuente I Vidaurreta I Responsiveness and clinically important differences for the WOMAC and SF-36 after hip joint replacement Osteoarthritis Cartilage 2005 13 1076 1083 10.1016/j.joca.2005.06.012 16154777

[B12] Cleeland C Osoba D Pain assessment in cancer Effect of cancer on quality of life 1991 Boca Raton: CRC Press 293 305

[B13] Keller S Bann CM Dodd SL Schein J Mendoza TR Cleeland CS Validity of the brief pain inventory for use in documenting the outcomes of patients with noncancer pain Clin J Pain 2004 20 309 318 10.1097/00002508-200409000-00005 15322437

[B14] Tan G Jensen MP Thornby JI Shanti BF Validation of the Brief Pain Inventory for chronic nonmalignant pain J Pain 2004 5 133 137 10.1016/j.jpain.2003.12.005 15042521

[B15] Mendoza T Mayne T Rublee D Cleeland C Reliability and validity of a modified Brief Pain Inventory short form in patients with osteoarthritis Eur J Pain 2006 10 353 361 10.1016/j.ejpain.2005.06.002 16051509

[B16] Williams VSL Smith MY Fehnel SE The Validity and Utility of the BPI Interference Measures for Evaluating the Impact of Osteoarthritic Pain J Pain Symptom Manage 2006 31 48 57 10.1016/j.jpainsymman.2005.06.008 16442482

[B17] Tyler EJ Jensen MP Engel JM Schwartz L The reliability and validity of pain interference measures in persons with cerebral palsy Arch Phys Med Rehabil 2002 83 236 239 10.1053/apmr.2002.27466 11833028

[B18] Gjeilo KH Stenseth R Wahba A Lydersen S Klepstad P Validation of the Brief Pain Inventory in Patients Six Months After Cardiac Surgery J Pain Symptom Manage 2007 34 648 656 10.1016/j.jpainsymman.2007.01.010 17629665

[B19] Bellamy N WOMAC Osteoarthritis Index: user guide V 2002 Queensland, Australia

[B20] Bellamy NB Validation study of WOMAC: a health status instrument for measuring clinically important patient relevant outcomes to antirheumatic drug therapy in patients with osteoarthritis of the hip or knee J Rheumatol 1988 15 1833 1840 3068365

[B21] Hays RD Sherbourne CD Mazel RM The RAND 36-Item Health Survey 1.0 Health Econ 1993 2 217 227 10.1002/hec.4730020305 8275167

[B22] Ware JE Jr Sherbourne CD The MOS 36-item short-form health survey (SF-36). I. Conceptual framework and item selection Med Care 1992 30 473 483 10.1097/00005650-199206000-00002 1593914

[B23] Ware JE Snow KK Kosinski M Gandek B SF-36 Health Survey Manual and Interpretation Guide 1993 Boston, MA, USA: New England Medical Center, The Health Institute

[B24] Klepstad P Loge JH Borchgrevink PC Mendoza TR Cleeland CS Kaasa S The Norwegian brief pain inventory questionnaire: translation and validation in cancer pain patients J Pain Symptom Manage 2002 24 517 525 10.1016/S0885-3924(02)00526-2 12547051

[B25] Radbruch L Loick G Kiencke P Lindena G Sabatowski R Grond S Validation of the German Version of the Brief Pain Inventory J Pain Symptom Manage 1999 18 180 187 10.1016/S0885-3924(99)00064-0 10517039

[B26] Cleeland Charles S The Brief Pain Inventory. User Guide 2009 Houston, Texas

[B27] Kirschner S Walther M Bohm D Matzer M Heesen T Faller H Konig A German short musculoskeletal function assessment questionnaire (SMFA-D): comparison with the SF-36 and WOMAC in a prospective evaluation in patients with primary osteoarthritis undergoing total knee arthroplasty Rheumatol Int 2003 23 15 20 12548437 10.1007/s00296-002-0253-4

[B28] Kapstad H Rustoen T Hanestad BR Moum T Langeland N Stavem K Changes in pain, stiffness and physical function in patients with osteoarthritis waiting for hip or knee joint replacement surgery Osteoarthritis Cartilage 2007 15 837 843 10.1016/j.joca.2007.01.015 17344069

[B29] Slatkowsky-Christensen B Kvien TK Bellamy N Performance of the Norwegian version of AUSCAN - a disease-specific measure of hand osteoarthritis Osteoarthritis Cartilage 2005 13 561 567 10.1016/j.joca.2005.02.013 15896986

[B30] Kelly KD Voaklander DC Johnston DW Newman SC Suarez-Almazor ME Change in pain and function while waiting for major joint arthroplasty J Arthroplasty 2001 16 351 359 10.1054/arth.2001.21455 11307134

[B31] Kiebzak GM Campbell M Mauerhan DR The SF-36 general health status survey documents the burden of osteoarthritis and the benefits of total joint arthroplasty: but why should we use it? Am J Manag Care 2002 8 463 474 12019598

[B32] Ostendorf M Buskens E van Stel H Schrijvers A Marting L Dhert W Verbout A Waiting for total hip arthroplasty: avoidable loss in quality time and preventable deterioration J Arthroplasty 2004 19 302 309 10.1016/j.arth.2003.09.015 15067641

[B33] Zelman DC Hoffman DL Seifeldin R Dukes EM Development of a metric for a day of manageable pain control: derivation of pain severity cut-points for low back pain and osteoarthritis Pain 2003 106 35 42 10.1016/S0304-3959(03)00274-4 14581108

[B34] Loge JH Kaasa S Hjermstad MJ Kvien TK Translation and performance of the Norwegian SF-36 Health Survey in patients with rheumatoid arthritis. I. Data quality, scaling assumptions, reliability, and construct validity J Clin Epidemiol 1998 51 1069 1076 10.1016/S0895-4356(98)00098-5 9817124

[B35] Cronbach LJ Coefficient alpha and the internal structure of tests Psychometrika 1951 16 297 334 10.1007/BF02310555

[B36] Fayers PM Machin D Quality of life: the assessment, analysis and interpretation of patient-reported outcomes 2007 second Chichester, UK: John Wiley

[B37] Wyrwich KW Bullinger M Aaronson N Hays RD Patrick DL Symonds T Estimating clinically significant differences in quality of life outcomes Qual Life Res 2005 14 285 295 10.1007/s11136-004-0705-2 15892420

[B38] Revicki D Hays RD Cella D Sloan J Recommended methods for determining responsiveness and minimally important differences for patient-reported outcomes J Clin Epidemiol 2008 61 102 109 10.1016/j.jclinepi.2007.03.012 18177782

[B39] SooHoo NF Vyas RM Samimi DB Molina R Lieberman JR Comparison of the Responsiveness of the SF-36 and WOMAC in Patients Undergoing Total Hip Arthroplasty J Arthroplasty 2007 22 1168 1173 10.1016/j.arth.2006.10.006 18078886

[B40] McHorney CA Tarlov AR Individual-patient monitoring in clinical practice: are available health status surveys adequate? Qual Life Res 1995 4 293 307 10.1007/BF01593882 7550178

[B41] Beaton DE Bombardier C Katz JN Wright JG A taxonomy for responsiveness J Clin Epidemiol 2001 54 1204 1217 10.1016/S0895-4356(01)00407-3 11750189

[B42] Tan G Jensen MP Thornby JI Shanti BF Validation of the Brief Pain Inventory for chronic nonmalignant pain J Pain 2004 5 133 137 10.1016/j.jpain.2003.12.005 15042521

